# Alcohol Drinking Obliterates the Inverse Association Between Serum Retinol and Risk of Head and Neck Cancer

**DOI:** 10.1097/MD.0000000000001064

**Published:** 2015-07-02

**Authors:** Ken-Chung Chen, Wei-Ting Hsueh, Chun-Yen Ou, Cheng-Chih Huang, Wei-Ting Lee, Sheen-Yie Fang, Sen-Tien Tsai, Jehn-Shyun Huang, Tung-Yiu Wong, Jiunn-Liang Wu, Chia-Jui Yen, Yuan-Hua Wu, Forn-Chia Lin, Ming-Wei Yang, Jang-Yang Chang, Hsiao-Chen Liao, Shang-Yin Wu, Jenn-Ren Hsiao, Chen-Lin Lin, Yi-Hui Wang, Ya-Ling Weng, Han-Chien Yang, Yu-Shan Chen, Jeffrey S. Chang

**Affiliations:** From the Department of Stomatology (K-CC, J-SH, T-YW), Department of Radiation Oncology (W-TH, Y-HW, F-CL, M-WY), Department of Otolaryngology (C-YO, C-CH, W-TL, S-YF, S-TT, J-LW, H-CL, J-RH, Y-SC), Division of Hematology and Oncology, Department of Internal Medicine (C-JY, J-YC, S-YW), and Department of Nursing, National Cheng Kung University Hospital, College of Medicine, National Cheng Kung University, Tainan, Taiwan (C-LL) and National Institute of Cancer Research, National Health Research Institutes, Tainan, Taiwan (J-YC, Y-HW, Y-LW, H-CY, JSC).

## Abstract

This analysis evaluated the association between serum retinol levels and risk of head and neck cancer (HNC) and whether the association is modulated by the use of alcohol, betel quid, or cigarette. In addition, we also examined the association between HNC risk and 2 single nucleotide polymorphisms, *TTR* rs1667255 and *RBP4* rs10882272, that have been associated with serum retinol levels. Unconditional logistic regression was performed to evaluate the association between serum retinol levels and HNC risk among 160 HNC cases and 198 controls. The associations between *TTR* rs1667255 and *RBP4* rs10882272 and serum retinol levels or HNC risk were evaluated by linear regression and unconditional logistic regression, respectively, for 418 HNC cases and 497 controls. The results showed that HNC cases had a lower mean serum retinol level compared with controls (845.3 μg/L vs 914.8 μg/L, *P* = 0.03). An inverse association between serum retinol levels and HNC risk occurred among never/occasional alcohol drinkers but not among regular drinkers. *TTR* rs1667255 was associated with serum retinol levels; however, neither *TTR* rs1667255 nor *RBP4* rs10882272 was associated with HNC risk. In summary, this study showed an inverse association between serum retinol levels and HNC risk, specifically among never/occasional alcohol drinkers. More studies are needed to establish the underlying biologic mechanisms for the inverse association between serum retinol levels and HNC risk and the modulation of this relationship by alcohol drinking.

## INTRODUCTION

Head and neck cancer (HNC), including cancers of oral cavity, oropharynx, hypopharynx, and larynx is one of the most common cancers in the world. Each year, approximately 599,637 patients with HNC are diagnosed worldwide.^1^ Alcohol, tobacco products, betel quid, and human papillomavirus (HPV; for cancer of the oropharynx) are the major established risk factors of HNC.^2–5^ Although, most of the HNC cases are caused by the consumption of alcohol, betel quid, and cigarette, the biological mechanisms underlying the development of HNC induced by alcohol, betel quid, and cigarette are not completely understood.

Vitamin A plays an important role in cell growth, differentiation, and apoptosis,^6^ and has been investigated for the use in HNC chemoprevention.^7^ The synthesis of the major active metabolite of vitamin A pathway, all-*trans* retinoic acid, is a 2-step process.^8^ In the first rate-limiting step, all-*trans* retinol is oxidized to all-*trans* retinal by several enzymes, including members of the cytosolic medium-chain alcohol dehydrogenases (ADHs) such as ADH1A, ADH1C, and ADH7 and members of the membrane-bound short-chain dehydrogenase/reductase.^8^ In the second step, all-*trans* retinal is converted to all-*trans* retinoic acid by several enzymes, including several acetaldehyde dehydrogenases (ALDHs) such as ALDH1A1, ALDH1A2, and ALDH1A3.^8^ All-*trans* retinoic acid exerts its action by binding to different retinoic acid receptors (RARs) and retinoid X receptors (RXRs), which are ligand-dependent transcription factors. Upon binding to all-*trans* retinoic acid, RARs and RXRs form heterodimers and bind to DNA sequences called RAR elements (RARE) or RXR elements (RXRE) in the promoter of target genes to induce the expression of these genes.^8^

Studies have suggested that the use of alcohol, cigarette, and betel quid may disrupt the metabolism or the function of retinoic acid. The metabolism of ethanol in alcoholic beverages may hinder the metabolism of retinoic acid by competitively inhibiting the conversion of retinol to retinoic acid, because the 2 pathways share many of the same enzymes.^6^ In addition, ethanol can induce the expression of cytochrome p450 2E1 to increase the catabolism of retinoic acid.^6^ Cigarette smoke contains at least 60 known human carcinogens, one of which is acetaldehyde, which can inhibit retinoic acid metabolism.^9^ In addition, cigarette smoke can generate reactive oxygen species to induce the expression of cytochrome P450 enzymes to increase the degradation of retinoic acid.^9^ Use of betel quid has been shown to suppress the expression of RAR-beta.^10^ These data suggested that at least some of the carcinogenic effects of alcohol, betel quid, and cigarette to induce the development of HNC may be due their abilities to disrupt the metabolism or the function of vitamin A. Investigating the interaction between vitamin A and alcohol, betel quid, or cigarette on the risk of HNC may further enhance our understanding regarding the development of HNC.

To date, only 2 studies have evaluated the association between serum vitamin A (in the form of retinol) and HNC risk.^11,12^ Furthermore, none has examined the interaction between vitamin A and alcohol, betel quid, or cigarette on the risk of HNC. The present study compared the serum retinol levels between HNC cases and controls. Additional analyses were performed to evaluate whether the association between serum retinol and HNC risk may be modulated by the consumption of alcohol, betel quid or cigarette. A previous genome-wide association study by Mondul et al reported that 2 single nucleotide polymorphisms (SNPs), *TTR* rs1667255 and *RBP4* rs10882272, are associated with serum retinol levels^13^; therefore, in addition to serum retinol, we also examined the association between these 2 SNPs and HNC risk.

## MATERIALS AND METHODS

This study was approved by the institutional review boards of the National Health Research Institutes (IRB number: EC1031001-E) and National Cheng Kung University Hospital (IRB number: B-ER-102–401). A signed informed consent was obtained from all study participants.

### Study Patients Recruitment

Recruitment of the study participants took place in the Department of Otolaryngology and the Department of Stomatology at the National Cheng Kung University Hospital from September 1, 2010 to November 12, 2013. Cases included patients who were diagnosed with squamous cell carcinoma of the head and neck, including oral cavity, oropharynx, hypopharynx, or larynx, who had not been diagnosed with other cancer previously, and were ages between 20 and 80 years. All of the HNC cases were confirmed by the Department of Pathology at the National Cheng Kung University Hospital. For comparison, control patients were selected by frequency-matching to cases on age (±5 years) and sex and comprised patients who received surgery for noncancerous conditions that are not related to the consumption of alcohol, betel quid, and cigarette and had never been diagnosed with cancer. Only men were included for the current analysis.

### Data Collection by Interview

Trained interviewers conducted an in-person interview with each study participant to ascertain information on education levels and consumption (frequency, amount, and duration) of alcohol, betel quid, and cigarette. Regular alcohol drinkers were defined as those who reported drinking at least once per week. Ever betel quid chewers were those who ever chewed betel quid daily for at least 6 consecutive months. Ever cigarette smokers were those who ever smoked at least 100 cigarettes during their lifetime. Former alcohol drinkers, betel quid chewers, and cigarette smokers were those who quit alcohol drinking, betel quid chewing, and cigarette smoking, respectively, for more than 6 months.

### Blood Sample Collection and Processing

For genotyping, peripheral blood was collected in a tube containing EDTA from each study participant. Buffy coat was collected by centrifuging the whole blood. A commercially available genomic DNA purification kit (Promega Corporation, Fitchburg, WI) was used to extract genomic DNA. Genomic DNA was stored in the −80°C refrigerator until ready to use.

For serum retinol measurement, blood was collected in a red-top serum tube wrapped in aluminum foil and stored in the 4°C refrigerator. The serum was then separated out on the same day by centrifugation. Serum was then stored in brown Eppendorf tubes to protect from light exposure. To minimize light exposure, the processing of serum was performed in a dark room. All serum samples were stored in the −80°C refrigerator until ready to use.

### Genotyping

Genotyping for *TTR* rs1667255 and *RBP4* rs10882272 was performed with the Taqman-based allelic discrimination method on an Applied Biosystems 7500 Real-Time Polymerase Chain Reaction System (Applied Biosystems, Foster City, CA).^14^ The primers and the probes for genotyping *TTR* rs1667255 and *RBP4* rs10882272 were included in commercially available kits ordered from Applied Biosystems (https://bioinfo.appliedbiosystems.com/genome-database/snp-genotyping.html). Several quality control measures were implemented to ensure the accuracy of the genotyping method. Ten percent of the DNA samples were randomly chosen for duplicate genotyping and the results were 100% concordant. The SNP call rate was 100% for *TTR* rs1667255 and 99.8% (913/915) for *RBP4* rs10882272. Both SNPs were in Hardy–Weinberg equilibrium. In addition, the minor allele frequencies (*TTR* rs1667255: frequency for C allele = 0.41; *RBP4* rs10882272: frequency for C allele = 0.10) of our control patients were very similar to those reported by the Hapmap database for Han Chinese from Beijing, China (*TTR* rs1667255: frequency for C allele = 0.37; *RBP4* rs10882272: frequency for C allele = 0.11).

### Serum Retinol Measurement

Serum retinol was measured by high performance liquid chromatography using a commercially available kit (Immunodiagnostik, Bensheim, Germany) according to the manufacturer's instruction.

### Statistical Analysis

The distributions of demographic variables and lifestyle habits (alcohol drinking, betel quid chewing, and cigarette smoking) were compared between cases and controls using *t* tests (for continuous variables) and Chi-squared tests (for categorical variables).

The levels of serum retinol were compared between HNC cases and controls using *t* test. Unconditional multivariable logistic regression was performed to estimate the odds ratio (OR) and 95% confidence interval (CI) of HNC risk associated with serum retinol levels. Serum retinol levels were analyzed as tertiles according to the distribution of serum retinol levels in control patients with the lowest tertile used as the referent group or were analyzed for the risk of HNC associated with every 100 μg/L increment. Three multivariable models were built with adjustment for: model 1: age; model 2: age and education; and model 3: age, education, and use of alcohol, betel quid, and cigarette, to evaluate the potential confounding effect of these covariates. Alcohol was adjusted as an ordinal variable according to the frequency of alcohol drinking (1 = never; 2 = 1–2 drinks per week; 3 = 3–5 drinks per week; and 4 = daily drinkers). Betel quid and cigarette were adjusted as continuous variables in pack-years. One pack-year of betel quid use = 1 pack of betel quid (20 betel quids) per day for 1 year. One pack-year of cigarette smoking = 1 pack of cigarette (20 cigarettes) per day for 1 year. To evaluate the influence of alcohol, betel quid, and cigarette on the association between serum retinol and HNC risk, unconditional logistic regression was performed stratified by the use of alcohol, betel quid, or cigarette.

To assess the association between serum retinol levels and the 2 SNPs (*TTR* rs1667255 and *RBP4* rs10882272), analysis of variance was performed to compare the mean serum retinol level by the number of the minor alleles (0, 1, or 2 copies). In addition, linear regression was performed to assess the level of serum retinol associated with every 1 copy of the minor allele.

Unconditional logistic regression was performed to estimate the OR and 95% CI for the association between *TTR* rs1667255 or *RBP4* rs10882272 and HNC risk using 3 different models: no mode of inheritance was assumed with those carrying 2 major alleles being the referent group; dominant model (the effect of carrying at least 1 copy of the minor allele); and log-additive model (an ordinal effect conferred by the minor allele). To evaluate the influence of alcohol, betel quid, and cigarette on the association between *TTR* rs1667255 or *RBP4* rs10882272 and HNC risk, unconditional logistic regression was performed stratified by the use of alcohol, betel quid, or cigarette.

## RESULTS

From September 1, 2010 to November 12, 2013, 418 men with HNC (273 oral cancers, 102 oro- and hypopharyngeal cancers, and 43 laryngeal cancers) and 497 men without HNC were successfully recruited with a participation of 77% and 86% for the cases and controls, respectively. Among them, 160 HNC cases and 198 controls had blood drawn for measuring serum retinol. Cases and controls were similar in age (*P* > 0.05) (Table 1 ). Controls had higher educational levels than cases. More cases than controls drank alcohol, chewed betel quid, and smoked cigarette. Patients who had serum retinol measurement were slightly younger than those without serum retinol measurement, but the consumption levels of alcohol, betel quid, and cigarette were generally similar between the 2 groups.

**TABLE 1:**
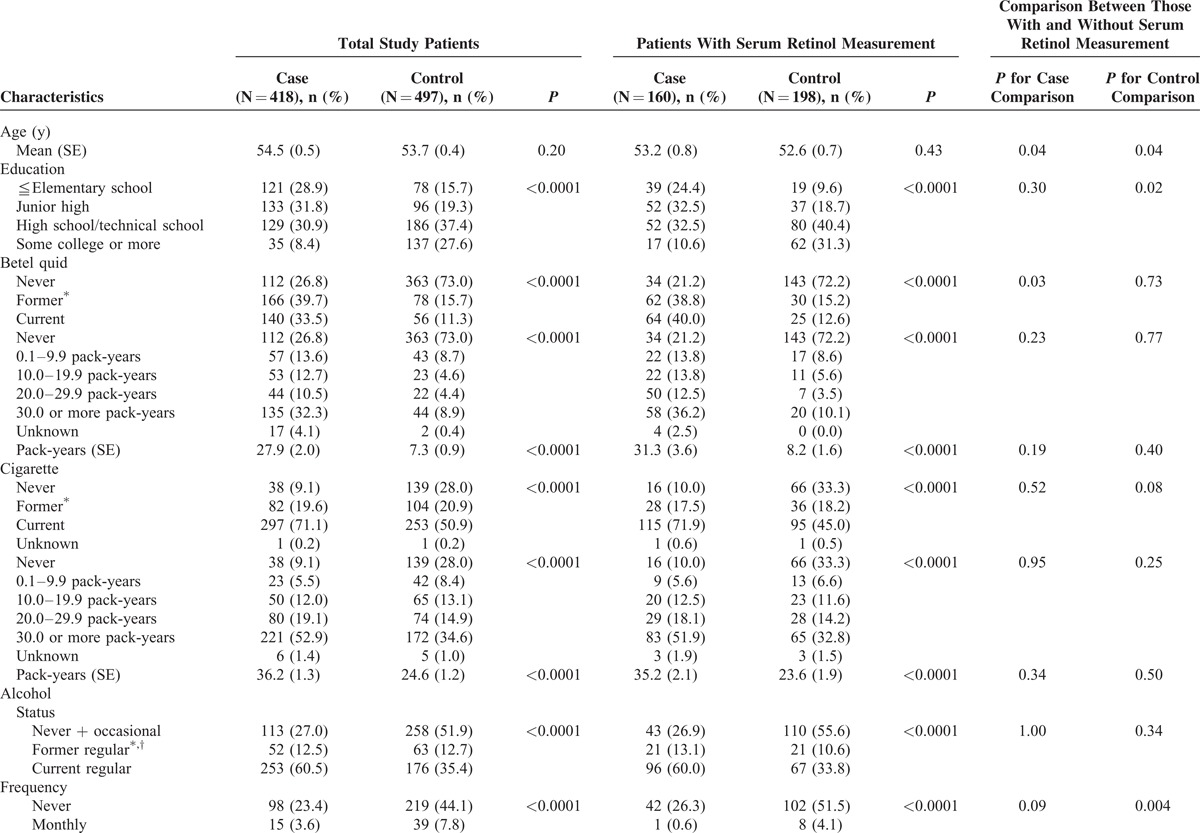
Demographic and Lifestyle Characteristics of the Head and Neck Cancer Patients and Control Patients

**TABLE 1 (Continued):**
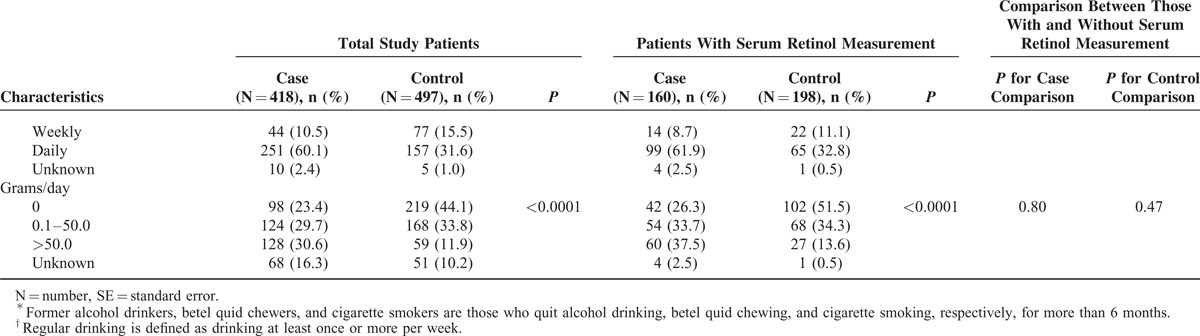
Demographic and Lifestyle Characteristics of the Head and Neck Cancer Patients and Control Patients

The mean serum retinol level of HNC cases was significantly lower than that of controls (845.3 μg/L vs 914.8 μg/L, *P* = 0.03). When examined in tertiles, the highest tertile of serum retinol level was associated with a reduced risk of HNC, although not statistically significant (Table 2). However, when examined as a continuous variable, every 100 μg/L increase in the level of serum retinol was associated with a statistically significant 8% reduction in HNC risk (OR = 0.92, 95% CI: 0.85–0.99). Adjusting for age, education, and the use of alcohol, betel quid, and cigarette did not change the OR by more than 10%, indicating that these covariates do not confound the inverse association between serum retinol levels and HNC risk.

**TABLE 2:**
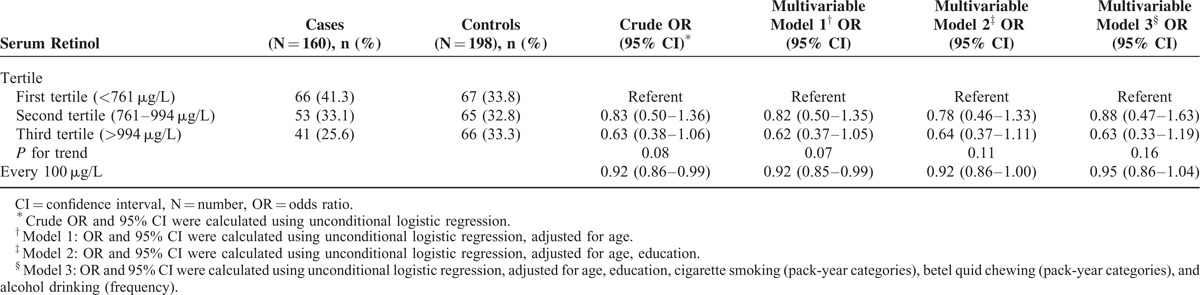
The Association Between Serum Retinol and Head and Neck Cancer

When stratified by alcohol drinking, the inverse association between serum retinol levels and HNC risk was only observed among never/occasional drinkers (those who drank less than once per week) and not among regular drinkers (Table 3). Among never/occasional drinkers, every 100 μg/L increase in serum retinol level was associated with a 17% reduction in HNC risk (OR = 0.83, 95% CI: 0.70–0.97). Compared with the lowest tertile of serum retinol level, the highest tertile was associated with a 73% reduction in HNC risk among never/occasional drinkers (OR = 0.27, 95% CI: 0.08–0.91). The association between serum retinol levels and HNC risk did not significantly differ by the use of betel quid or cigarette.

**TABLE 3:**
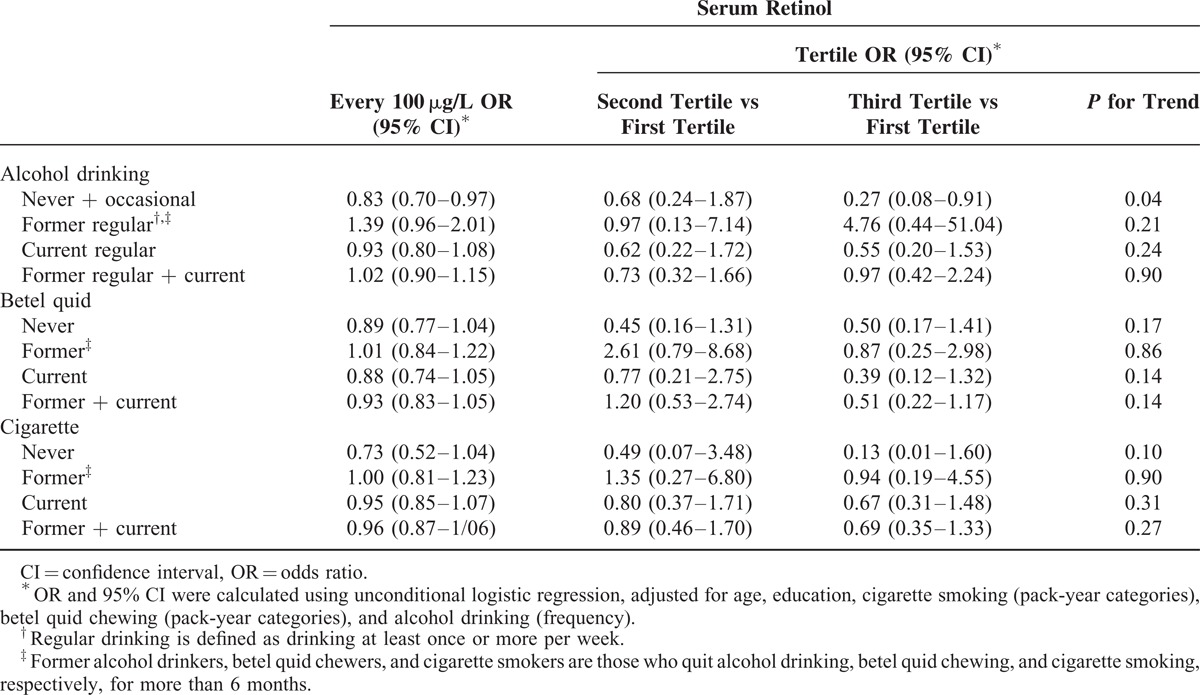
The Association Between Serum Retinol and Risk of Head and Neck Cancer Stratified by the Use of Alcohol, Betel Quid, or Cigarette

The level or serum retinol increased significantly with the number of minor alleles for *TTR* rs1667255 among controls. For every 1 copy of minor allele C of *TTR* rs1667255, the level of serum retinol increased significantly by 59.8 μg/L (*P* = 0.04). In contrast, serum retinol levels were not associated with *TTR* rs1667255 among HNC cases. *RBP4* rs1088227 was not associated with serum retinol levels among either cases or controls (Table 4).

**TABLE 4:**
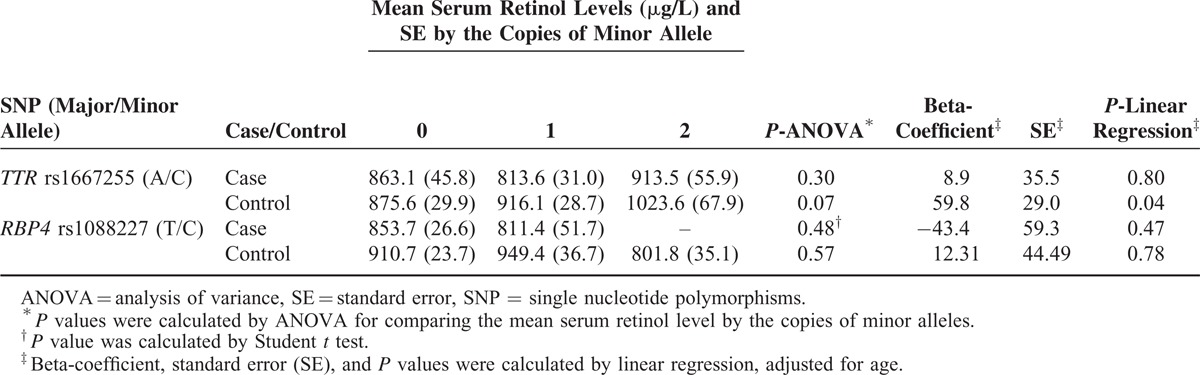
The Association Between *TTR* rs1667255 or *RBP4* rs10882272 and Serum Retinol Level

Neither *TTR* rs1667255 nor *RBP4* rs1088227 was associated with HNC risk (Table 5). Even after stratified by alcohol drinking, betel quid chewing, and cigarette smoking, no significant association between *TTR* rs1667255 or *RBP4* rs1088227 and HNC risk was observed.

**TABLE 5:**
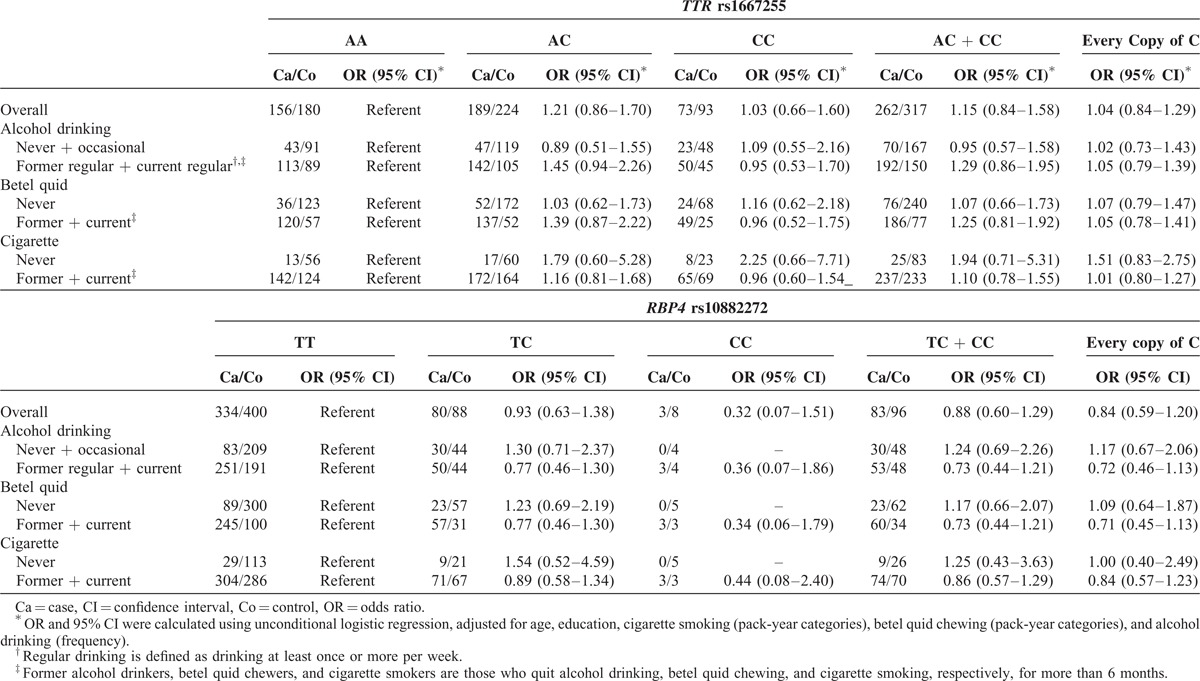
The Association Between *TTR* rs1667255 or *RBP4* rs10882272 and Risk of Head and Neck Cancer, Overall and Stratified by the Use of Alcohol, Betel Quid, or Cigarette

## DISCUSSION

In the present study, we observed a lower level of serum retinol among HNC cases compared with controls. The inverse association between serum retinol levels and HNC risk disappeared in the presence of alcohol drinking. We also confirmed the result of a previous GWAS that reported an association between *TTR* rs1667255 and serum retinol levels.^13^ However, we did not find an association between *TTR* rs1667255 and HNC risk.

The inverse association between serum retinol levels and HNC risk from the present study is consistent with 1 of the only 2 published studies on the association between serum retinol and HNC risk.^11,12^ In a case–control study (120 cases and 120 controls) with 4 ethnic groups (Malay, Chinese, Indian, and Indigenous) from Malaysia, Athirajan et al observed that higher levels of serum retinol were associated with a reduced risk of oral cancer across all 4 ethnic groups, although the strength of association varied by ethnic groups, with the association among Indians showing the strongest statistical significance.^12^ In contrast, a nested case–control study (28 cases and 112 controls from a cohort of 25,802 adults from Washington County, MD) by Zheng et al did not observe a significant association between serum retinol levels and risk of developing oral and pharyngeal cancer.^11^ The inconsistencies between studies may be explained by several reasons. First, the association between serum retinol levels and HNC may differ by ethnic groups possibly due to the influence of lifestyle factors. For example, our study showed that the inverse association between serum retinol levels and HNC risk only occurred among nondrinkers. It is possible that the association between serum retinol levels and HNC risk may depend on the prevalence of alcohol drinking among different ethnic groups. Because our study is the only 1 that has ever evaluated the modulation of the association between serum retinol levels and HNC risk by alcohol drinking and other lifestyle factors (betel quid chewing and cigarette smoking), more studies are needed to confirm this hypothesis. Another reason for the inconsistent results across studies may be the difference in study design. Our study and the study by Athirajan et al^12^ both used case–control study design, which may suffer from reverse causality, since the blood samples for the HNC cases were taken at the time of diagnosis and the level of serum retinol could have been affected by the oncogenic process of HNC. Study by Zheng et al used a nested case–control study design where the blood samples were taken before the occurrence of HNC and therefore a temporal relationship could be more clearly established.^11^ However, Zheng et al only included 28 cases and 112 controls and thus their results could more likely be affected by random variations and a lack of statistical power.^11^ More studies with a larger sample size and a cohort or nested case–control study design are needed to confirm the inverse association between serum retinol levels and HNC risk.

The inverse association between serum retinol levels and HNC risk implies that higher serum retinol levels may be protective against the development of HNC. Retinoids, which are vitamin A-related compounds, have been shown to possess antiproliferative and antioxidant activities and may promote differentiation or apoptosis of cancer cells.^15^ Because of these properties, retinoids have been tested for their chemopreventive efficacy in various cancers, including HNCs; however, the results of the clinical trials for the prevention of HNC have been disappointing.^16,17^ This indicates that the role of vitamin A in the oncogenesis of HNC is not completely understood and more investigations are needed to understand the utility of retinoids for the chemoprevention of HNC.

Our analysis showed that the inverse association between serum retinol levels and HNC risk only existed among never/occasional alcohol drinkers and such association was not observed among regular alcohol drinkers. This suggested that the protective effect of vitamin A against HNC may be inhibited by the use of alcohol. The biologically active compound of vitamin A is retinoic acid, which is produced from retinol in a 2-step process (step 1: retinol to retinal; step 2: retinal to retinoic acid).^6^ The metabolism of ethanol in alcoholic beverage shares the similar 2-step process (step 1: ethanol to acetaldehyde; step 2: acetaldehyde to acetic acid) to the metabolism of retinol. Ethanol may compete with retinol for the same metabolic enzymes, including ADHs and ALDHs to reduce the production of retinoic acid.^6^ Alcohol consumption may also induce the production of cytochrome P450 2E1 (CYP2E1) to increase the catabolism of retinoic acid.^6^ The decreased production of retinoic acid due to alcohol consumption may ultimately result in the decreased expression of retinoid-responsive genes.^6^ It is interesting to note that *ALDH2*, an ethanol metabolizing gene that codes for aldehyde dehydrogenase responsible for converting the carcinogenic acetaldehyde to the noncarcinogenic acetic acid, also contains retinoid receptor response element in the promoter region. Alcohol may not only induce the oncogenesis of HNC by inhibiting the generation of retinoic acid to affect cell growth, differentiation, and apoptosis, the reduced production of retinoic acid may also lead to the decreased expression of *ALDH2* to slow down the clearance of the carcinogenic acetaldehyde produced by the ethanol metabolism.

Our study confirmed the association between the C allele of *TTR* rs1667255 and the increased serum retinol levels as reported by Mondul et al.^13^ In contrast, we did not observe a significant association between serum retinol levels and the minor C allele of *RBP4* rs1088227. The much lower minor allele frequency (10%) and the lower percentage (1.6%) of CC genotype of *RBP4* rs1088227 in our study population compared with the minor allele frequency (35%) in the study population of Mondul et al^13^ may have reduced the statistical power in our study to detect a significant association between *RBP4* rs1088227 and serum retinol levels. Even though our study showed a significant association between *TTR* rs1667255 and serum retinol levels, there was no significant association between *TTR* rs1667255 and HNC risk. It is possible that *TTR* rs1667255 only contributes to part of the variation in serum retinol levels. The serum retinol level is likely determined by a complex interaction between genetic and environmental factors. Therefore, an association between *TTR* rs1667255 and serum retinol levels may not necessarily translate into an association between *TTR* rs1667255 and HNC risk.

This study has several limitations. First, we only studied the association between HNC risk and serum retinol. More studies are needed to comprehensively investigate the role of retinoids, including the metabolites of retinol (retinal and retinoic acid), in the development of HNC. Second, although HPV is an established risk factor for oropharyngeal cancer.^18^ We did not have access to tumor samples to test for the presence of HPV DNA in the tumor tissues. However, HPV only plays a major role in the development of oropharyngeal cancer, which accounts for only 13% of our HNC cases. Therefore, we believe that HPV did not play a major role to confound the association between serum retinol and HNC risk in our study. Third, because the blood sample collection for serum retinol, which requires special handling to avoid light exposure, was initiated later in the study, we were only able to measure serum retinol in 160 HNC cases and 198 controls. The measurement of serum retinol in a subset of study patients may have lead to selection bias. However, those with and without serum retinol measurement were similar in the consumption levels of alcohol, betel quid, and cigarette and thus, we believe that the selection bias was not a major problem for the current analysis. Finally, although our sample size of 160 HNC cases and 198 controls are not considered a large sample size, it is the largest study to date on the association between serum retinol and HNC risk. In addition, our findings regarding the inverse association between serum retinol levels and HNC risk and the disruption of this association by alcohol drinking have strong biological basis. However, for any epidemiologic finding, replication is the key. Future studies with a large sample size are needed to confirm the results of our study.

This study has a couple strengths. First, this is 1 of the only 3 studies that have examined the association between serum retinol and the risk of HNC. Even though more studies are needed to understand the role of vitamin A in the development of HNC, results generated from our study and others have contributed toward this aim. Second, this is the first study to examine the influence of alcohol, betel quid, and cigarette on the association between serum retinol levels and HNC risk. The results of this study provided additional information regarding the carcinogenicity of alcohol in the development of HNC.

In summary, our study showed that increased levels of serum retinol were associated with a lower HNC risk. The inverse association between serum retinol levels and HNC risk was not observed among alcohol drinkers. More studies are needed to establish the underlying biologic mechanisms for the inverse association between serum retinol levels and HNC and the modulation of this relationship by alcohol drinking.
